# Comparison of Transverse Island Flap Onlay and Tubularized Incised-Plate Urethroplasties for Primary Proximal Hypospadias: A Systematic Review and Meta-Analysis

**DOI:** 10.1371/journal.pone.0106917

**Published:** 2014-09-08

**Authors:** Dongdong Xiao, Xin Nie, Wenyue Wang, Juan Zhou, Ming Zhang, Zhe Zhou, Yang Zhao, Meng Gu, Zhong Wang, Mujun Lu

**Affiliations:** 1 Department of Urology, Shanghai Ninth People’s Hospital, Shanghai Jiao Tong University, School of Medicine, Shanghai, People’s Republic of China; 2 Department of General Surgery, Shanghai Ninth People’s Hospital, Shanghai Jiao Tong University, School of Medicine, Shanghai, People’s Republic of China; Sapienza, University of Rome, School of Medicine and Psycology, Italy

## Abstract

**Purpose:**

This meta-analysis was conducted to compare postoperative outcomes between transverse island flap (TVIF) onlay and tubularized incised-plate (TIP) urethroplasties for primary proximal hypospadias.

**Materials and Methods:**

A comprehensive literature search updated to 21^st^ May 2014 was carried out for relevant studies. After literature identification and data extraction, odds ratio (OR) with 95% confidential interval (CI) was calculated to compare postoperative complication rate between TVIF onlay and TIP. Meta-regression and subgroup analyses were applied to find potential affective factors.

**Results:**

A total of 6 studies including 309 patients receiving TVIF onlay and 262 individuals subjected to TIP met inclusion criteria. The synthetic data suggested that TVIF onlay and TIP were comparable in terms of total complication rate (OR 0.85, 95% CI 0.56–1.30, p = 0.461), fistula (OR 0.68, 95% CI 0.38–1.21, p = 0.194), recurrent curvature (OR 1.16, 95% CI 0.43–3.12, p = 0.766), dehiscence (OR 0.95, 95% CI 0.33–2.74, p = 0.920), diverticulum (OR 1.90, 95% CI 0.53–6.78, p = 0.321), meatal stenosis (OR 0.74, 95% CI 0.20–2.77, p = 0.651) and urethral stricture (OR 1.49, 95% CI 0.41–5.50, p = 0.545), without significant heterogeneity for each comparison group. Meta-regression and subgroup analyses revealed no significant findings. One-way sensitivity analysis indicated that the results were stable. No publication bias was detected using both funnel plot and Egger’s test. Also, there were no obvious differences observed in cosmetic and functional outcomes.

**Conclusions:**

This meta-analysis suggests that TVIF onlay and TIP urethroplasties are clinically equivalent. Given the inherent limitations of included studies, this conclusion should be interpreted with caution and wait to be confirmed by more well-designed randomized controlled trials with high quality in the future.

## Introduction

Hypospadias has been treated with more than 200 different surgical approaches, such as tubularized incised-plate (TIP) [1], transverse island flap (TVIF) onlay [2], tubularized preputial flap [3], vertical preputial island flap [4], vertical preputial flap with double skin island [5] and so on.

To select an optimal urethroplasty technique for hypospadias is always a challenge, because numerous factors need to be taken into consideration. Apart from chordee severity, adequate tissue for urethral reconstruction, urethral plate quality and surgeons’ experience and preference, it is well-known that the initial meatal location affects the choice of urethroplasty technique and the prognosis of hypospadias repair to a large extent [6]. In addition, the necessity of glanuloplasty and preputioplasty also needs consideration [7].

Originally introduced for distal hypospadias, TVIF onlay [2], a variation of tubularized preputial flap, which was first reported by Standoli et al [8] and Duckett et al [3], and TIP, invented by Snodgrass et al [1], have extended their effectiveness to proximal hypospadias [9], [10]. Accumulating studies have been comparing the postoperative outcomes between these two techniques for proximal hypospadias, however, with inconsistent results. Thus, this meta-analysis was conducted to make a more precise comparison of the postoperative outcomes between TVIF onlay and TIP urethroplasties for primary proximal hypospadias.

## Materials and Methods

### Search Strategy

A comprehensive literature search updated to 21^st^ May 2014 was conducted in Pubmed, EMASE and the Cochrane Library using the keywords related to TVIF onlay in combination with TIP, with language restricted to English only. In addition to compute-based searches, scanning of the bibliographies of relevant articles and examination of reviews in this field, potential eligible comparative studies including TVIF onlay and TIP were carefully sought. Only published articles with full-text were included. This meta-analysis was performed according to PRISMA statement [11].

### Inclusion Criteria

The inclusion criteria were as follows: a) comparative study of the prognosis among urethroplasties for primary proximal hypospadias repair; b) including both TVIF onlay and TIP; c) patients subjected to urethroplasties before adolescence (<10 years old); d) sufficient published data for estimating an odds ratio (OR) with 95% confidence interval (CI). Publications such as reviews, surveys, replies, comments and protocols were excluded. If same population existed in more than one studies, the most recent and complete one was included.

### Definitions

The TVIF onlay and TIP urethroplasties performed in included studies was originated from the description of Elder et al [2] in 1987 and Snodgrass et al [1] in 1994, respectively. In order to reduce clinical heterogeneity, only standard TVIF onlay and TIP were included in this analysis. Dorsal plication was applied if patients presented severe penile ventral curvature.

The definitions of postoperative outcomes were accorded with authors’ descriptions, for they were seldom defined, especially for subjective outcomes like cosmetics. Generally, a successful hypospadias repair was defined as having a functional urethra with normal stream, without fistula, diverticulum, stricture or other postoperative complications, and having a normal looking straight penis with a conically shaped glans and a slit-like meatus at its tip.

### Data Extraction and Quality Assessment

Main characteristics of each eligible study and detailed data of postoperative outcomes of TVIF onlay and TIP were carefully extracted according to a predefined protocol. For all included studies were cohort studies, the quality of them was assessed by the Newcastle-Ottawa Quality Assessment Scale (NOS) [12] and Levels of Evidence [13]. These processes were carried out carefully and independently by two authors: D.D. Xiao and M.J. Lu. Disputes were resolved by discussion and consultation to another author Z Wang, in which final decision was made by a majority vote.

The data from included studies were not all fully presented. Some studies combined proximal hypospadias with middle or distal cases. Frequencies of postoperative complications of TVIF onlay and TIP were sometimes presented collectively. Moreover, preoperative accompanied disorders and details of surgical procedures were not always available. If necessary, corresponding authors of each included study were contacted to ask for missing data needed in this meta-analysis.

### Statistical Analysis

The pooled OR with 95% CI was calculated to compare the postoperative complication rate between TVIF onlay and TIP. The significance of the pooled OR was determined by the Z-test, and was considered as statistically significant if a p-value<0.05. Evaluated by *Chi*-square test, heterogeneity was considered significant if a p-value<0.1 [14]. For the relatively small sample size and potential clinical heterogeneity between studies, random-effects model (DerSimonian and Laird method) was used [15]. One-way sensitivity analysis was used to assess the stability of our results, namely, a single study was deleted each time to reflect the influence of the individual data set to the pooled OR [16].

Begg’s funnel plot and Egger’s test were performed to examine potential publication bias. Obvious asymmetry of funnel plot means evident publication bias [17]. The significance of the intercept was determined by the t-test suggested by Egger, in which case p<0.05 was considered statistically significant [18]. Furthermore, meta-regression and subgroup analyses were performed to search for potential affective factors of postoperative complication rate of TVIF onlay and TIP.

All calculations were conducted using the Stata version 12.0 (StataCorp, College Station, Texas). All p-values were two sided.

## Results

### Study Selection

**Figure 1:**
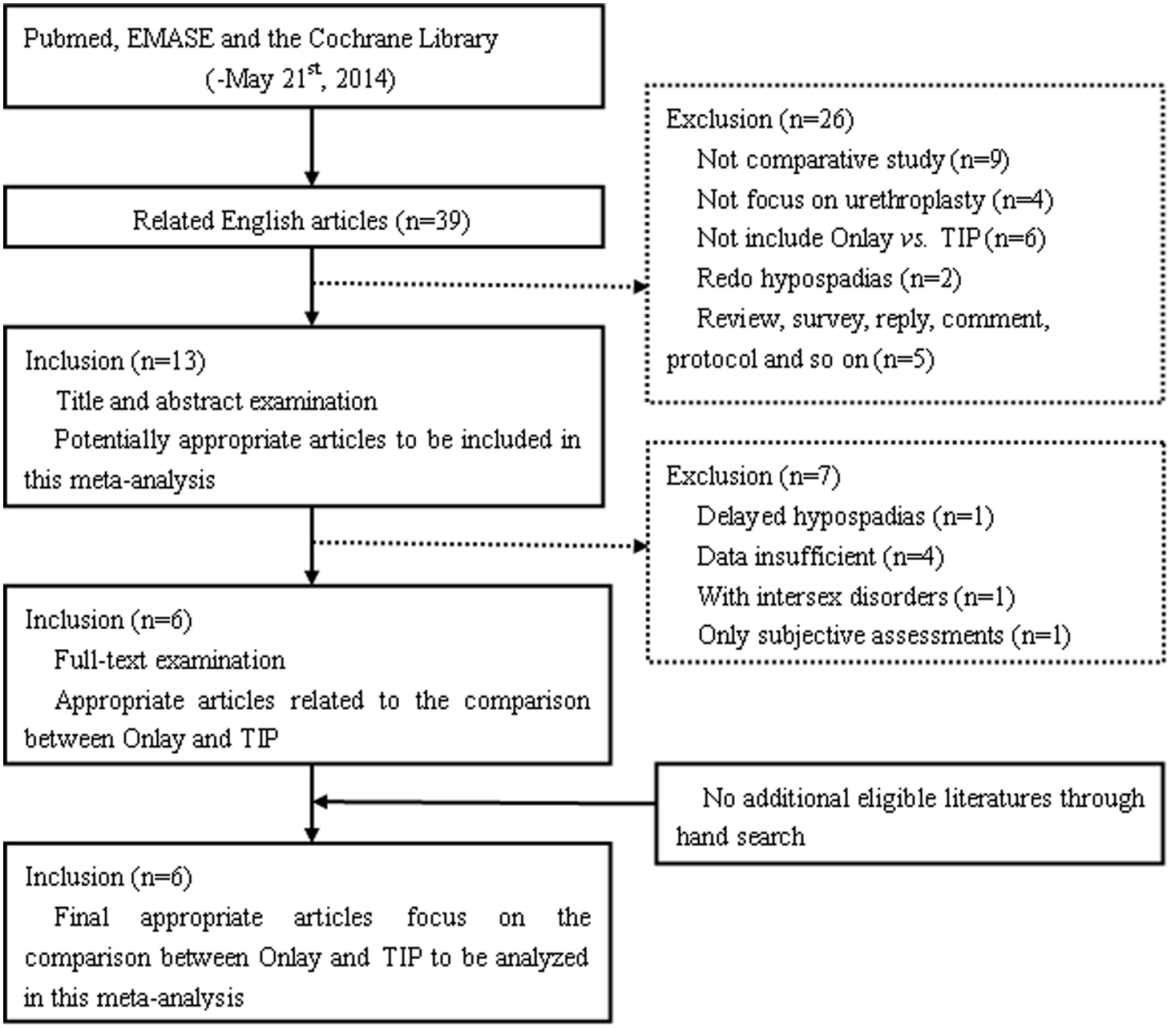
Flow diagram of eligible literatures identification from different medical databases.

39 related literatures were retrieved to be screened thoroughly through the identification process (Figure **1**). After title, abstract and full-text examination according to the inclusion criteria, a total of 6 studies with 309 patients receiving TVIF onlay and 262 individuals subjected to TIP were included in this meta-analysis.

### Study Characteristics

Main characteristics of included studies were presented in Table **1**. Designed as retrospective cohort studies, all included studies presented relatively high quality, with the NOS scores ranging from 8 to 9 and the Level of Evidence scored 2b. Summary of surgical procedures (Table **2**) demonstrated that except for neourethra formation and missing data, there was no obvious variation of surgical procedures between TVIF onlay and TIP conducted in each comparative study. The frequencies of preoperative treatment and accompanied disorders were presented in Table **3**. It was noteworthy that in one study (2007 Braga [19]), patients receiving TVIF onlay had a significantly higher rate of severe ventral curvature than TIP patients.

**Table 1:** Main characteristics of included studies.

Publication	Studydesign	Region	No. ofpatients(Onlay/TIP)	Mean age ofpatients(Onlay/TIP)	Meanfollow-upduration(Onlay/TIP)	Preoperativetestosterone	Hypospadias location				Quality evaluation	
							Proximalpenile	Penoscrotal	Scrotal	Perineal	NOS	Level ofevidence
2014Xu	Retrospective cohort study	China	93	4.5±3.3 y	25 (14–51) m	0	59	34	0	0	8	2b
			83	4.1±3.0 y	22 (12–48) m	0	50	33	0	0		
2013 Castagnetti	Retrospective cohort study	Italy	31	NA	6.9 (3–8.4) y	12	0	23	8		8	2b
			26		4.2 (2.2–8.3) y	9	0	21	5			
2012Prat	Retrospective cohort study	Israel	68	2.7±3.9 y	NA^a^	NA	NA	NA	NA	NA	9	2b
			6									
2010Moursy	Retrospective cohort study	Egypt	57	15.3±6.035 m	33.6±8.703 m	0	29	24	4	0	8	2b
			96	14.9±5.306 m	33.9±9.91 m	0	54	27	15	0		
2009 Sujijantararat	Retrospective cohort study	Thailand	20	48 (9–132) m	40 m	0	0	20	0	0	8	2b
			16	49 (10–348) m	32 m	0	0	16	0	0		
2007Braga	Retrospective cohort study	Canada	40	17.8 (10–58) m	38.8 (16–80) m	10	0	40	0	0	8	2b
			35	17 (9–91) m	30 (6–74) m	12	0	35	0	0		

a All patients underwent a routine follow-up examination at 6 and 12 months postoperatively.

**Table 2:** Summary of surgical procedures.

Publication	Detailed surgicaltechnique	Coverage of the neourethra	Suture for urethroplasty	Catheter size	Catheter duration	Suture forglansplasty or theremaining skin	Urinary diversion
2014Xu	Standard Onlay	Adjacent tissues &dartos pedicle	5–0 Vicrylrunning suture	6–12 F	NA	NA	NA
	Standard TIP						
2013Castagnetti	Standard Onlay	NA	6–0 absorbablesutures	6–8 F siliconecatheter	9 d	NA	NA
	Standard TIP						
2012Prat	Standard Onlay	NA	NA	NA	NA	NA	Silastic stentsfor 24 h to 6–7 d
	Standard TIP						
2010Moursy	Standard Onlay	Subcutaneous tissue	6–0 Vicrylsutures	10 Ch urethralcatheter	8 d	6–0 Vicrylsutures	Suprapubic cystocathfor 14 d
	Standard TIP						
2009Sujijantararat	Standard Onlay	Vascularized dartos flapfrom dorsal penile skin	6–0 Monocrylrunning suture	6 F nasogastrictube	NA	6–0 Vicrylinterruptedsutures	NA
	Standard TIP						
2007Braga	Standard Onlay	Dartos flap &spongiosal tissue	7–0 polydioxanone running suture	8 F feedingtube	10.1 (7–14)^a^	NA	8 Fr silastic stentor an 8 Fr Foleycatheter for 7–14 d
	Standard TIP	Vascular pedicle			8.8 (7–10)^a^		

a Mean catheter duration was not significantly different between both Onlay and TIP.

**Table 3:** Preoperative accompanied disorders and postoperative complications of included studies.

Publication	Surgicaltechnique	No. ofPatients	Dorsalplication	Preoperative accompanied disorders					Postoperative complications						
				Chordee	Penoscrotaltransposition	Bifidscrotum	Congenitalhernia	Undescendedtestis	Total	Fistula	Recurrentcurvature	Dehiscence	Diverticulum	Meatalstenosis	Urethral stricture
2014Xu	Onlay	93	48	53	2	3	2	3	20	10	3	0	4	1	2
	TIP	83	39	45	1	2	2	5	15	8	4	2	0	1	0
2013Castagnetti	Onlay	31	23	31	NA	NA	NA	NA	5	NA	NA	NA	NA	NA	NA
	TIP	26	18	26	NA	NA	NA	NA	7	NA	NA	NA	NA	NA	NA
2012Prat	Onlay	68	NA	NA	NA	NA	NA	NA	32	NA	NA	NA	NA	NA	NA
	TIP	6	NA	NA	NA	NA	NA	NA	3	NA	NA	NA	NA	NA	NA
2010Moursy	Onlay	57	NA	NA	0	4	0	0	8	4	0	3	0	1	0
	TIP	96	NA	NA	0	6	0	0	13	8	0	2	0	3	0
2009Sujijantararat	Onlay	20	NA	NA	NA	NA	NA	NA	6	4	0	0	2	0	0
	TIP	16	NA	NA	NA	NA	NA	NA	6	4	0	1	1	0	0
2007Braga	Onlay	40	27	NA	16	NA	NA	NA	18	8	5	2	0	1	2
	TIP	35	19	NA	14	NA	NA	NA	21	15	2	3	0	1	0

### Meta-analysis Results

**Figure 2:**
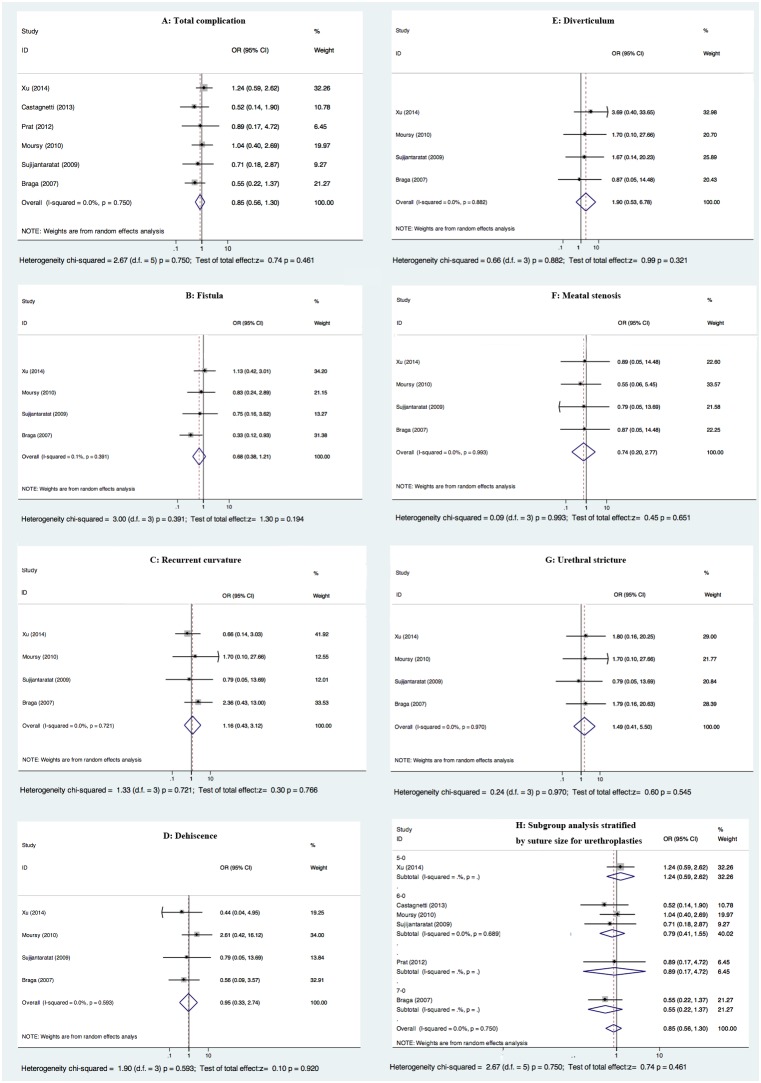
Forest plots of postoperative complications between TVIF onlay and TIP.

It was suggested that TVIF onlay and TIP were equivalent for primary proximal hypospadias in terms of total complication rate (OR 0.85, 95% CI 0.56–1.30, p = 0.461, Figure **2A**). Two studies (2014 Xu [20] and 2010 Moursy [21]) reported higher total complication rate of TVIF onlay than TIP, while the other four studies [19], [22]–[24] derived the opposite results. But no study showed significant difference in total complication rate between two techniques.

Four studies [19]–[21], [24] described each separate complication in detail, while the remaining two studies (2013 Castagnetti [22] and 2012 Prat [23]) still lacked sufficient complication data after contacting the corresponding authors.

Fistula was demonstrated to occur more frequently in patients receiving TIP in most studies [19], [21], [24], including one study (2007 Braga [19]) with significant difference. But the synthetic result of fistula came with no statistical difference (OR 0.68, 95% CI 0.38–1.21, p = 0.194, Figure **2B**). As for recurrent curvature, two studies (2010 Moursy [21] and 2009 Sujijantararat [24]) reported no recurrent curvature cases. One study (2014 Xu [20]) reported a non-significantly higher rate after TIP, while another (2007 Braga [19]) revealed the opposite result. The pooled data showed no significant difference between two techniques (OR 1.16, 95% CI 0.43–3.12, p = 0.766, Figure **2C**). In the cases of dehiscence, most studies [19], [20], [24] reported that it was more frequent in TIP, but without statistical difference. No significance was observed when combining the four studies (OR 0.95, 95% CI 0.33–2.74, p = 0.920, Figure **2D**). Two studies (2010 Moursy [21] and 2007 Braga [19]) reported no diverticulum cases. It was indicated that the incidence rate of diverticulum had no significant difference (OR 1.90, 95% CI 0.53–6.78, p = 0.321, Figure **2E**). One study (2009 Sujijantararat [24]) reported no meatal stenosis cases. A higher rate of meatal stenosis for TIP was observed in the rest three studies, but without significant difference, as same as the synthetic result (OR 0.74, 95% CI 0.20–2.77, p = 0.651, Figure **2F**). Without exception, no significant difference was observed in urethral stricture cases (OR 1.49, 95% CI 0.41–5.50, p = 0.545, Figure **2G**), which were not reported in two studies (2010 Moursy [21] and 2009 Sujijantararat [24]).

It was proved by the Q-test that no heterogeneity existed in each comparison (Figure **2**). Similarly, no single study influenced the pooled OR qualitatively as indicated by sensitivity analyses, demonstrating that the results were stable.

### Additional Analysis

Meta-regression analysis suggested that mean age of patients (coefficient −0.008, p = 0.647), mean follow-up duration (coefficient 0.008, p = 0.758) and the percentage of proximal penile and penoscrotal sites (coefficient 1.196, p = 0.505) were not the affective factors for the comparison between TVIF Onlay and TIP for primary proximal hypospadias.

Subgroup analysis was carried out stratified by suture size for urethroplasties, among which no significant difference was indicated in 5–0 suture (OR 1.24, 95% CI 0.59–2.62, p = 0.569), 6–0 suture (OR 0.79, 95% CI 0.41–1.55, p = 0.497) and 7–0 suture (OR 0.55, 95% CI 0.22–1.37, p = 0.196) subgroups (Figure **2H**).

For inadequate studies describing preoperative testosterone injection, dorsal placation, preoperative accompanied disorders and other surgical procedure factors, meta-regression or subgroup analysis was not conducted for them.

### Publication Bias

**Figure 3:**
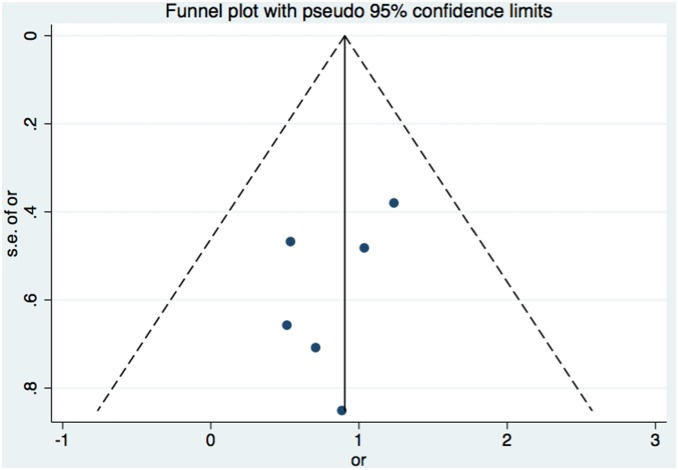
Funnel plot of publication bias for total complication.

Both Begg’s funnel plot and Egger’s test were used to evaluate the publication bias of the literatures. No evidence of obvious asymmetry was observed (Figure **3**). Egger’s test also revealed that there was no significant publication bias (p = 0.351).

### Cosmetic and Functional Results

In addition to general assessments, two studies (2014 Xu [20] and 2013 Castagnetti [22]) applied pediatric penile perception score (PPPS) to evaluate postoperative cosmetic outcomes of TVIF onlay and TIP, with no significant difference observed, among which one study (2013 Castagnetti [22]) also evaluated urinary symptoms, in which no statistical difference was observed either (Table **4**).

**Table 4:** Cosmetic and functional evaluations of included studies.

Publication	Surgical technique	No. ofPatients	Evaluations otherthan generalassessments	PPPS						
				Penilelength	Meatus	Glans shape	Penile skin	Penileaxis	Generalappearance	Total
2014Xu	Onlay	93	PPPS	2 (1–2)	2 (1–3)	2 (2–3)	2 (2–3)	2 (1–3)	2 (2–3)	11 (10–15)
	TIP	83		2 (1–2)	2 (2–3)	2 (2–3)	2 (2–3)	2 (1–3)	2 (2–3)	12 (10–15)
2013Castagnetti	Onlay	31	PPPS &UrinarySyptoms	1 (1–2)	2 (2–2)	2 (2–2.5)	2 (1–2)	2 (1–2)	2 (2–2)	11 (10–12)
	TIP	26		2 (1–2)	2 (2–3)	2 (2–2)	2 (2–2)	2 (1–2)	2 (2–2)	11 (11–12)
2012Prat	Onlay	68	NA							
	TIP	6								
2010Moursy	Onlay	57	Uroflowmetry							
	TIP	96								
2009Sujijantararat	Onlay	20	NA							
	TIP	16								
2007Braga	Onlay	40	Uroflowmetry							
	TIP	35								

Two studies (2010 Moursy [21] and 2007 Braga [19]) used uroflowmetry to assess postoperative function outcomes (Table **4**). The former study reported 54 (94.74%) TVIF Onlay and 91 (94.79%) TIP patients showed normal cosmetic and functional outcomes, without significant difference between groups. The latter one revealed that mean average flow rates and mean peak flow rates of TIP were significantly lower than TVIF onlay at a mean age of 5.1 years, with plateau-shaped prolonged uroflow curve happening more frequently in TIP, while post-void residual greater than 10% of voided volume was not statistically different.

## Discussion

Based on anatomical studies of urethral plate in hypospadias patients, various components were reported to be present in normal urethral spongiosum [25]. Nowadays, particular importance has been attached to urethral plate preservation [26], a vascular tissue rich in nervous supply and muscular backing, which is considered extremely suitable for urethroplasty.

Both TVIF onlay and TIP urethroplasties employ urethral plate as a crucial constituent of neourethra formation and come up with favorable outcomes. Given the relative simplicity of operative procedures, low complication rate and acceptable cosmetic appearance in distal hypospadias, TIP has been gradually applied to more proximal hypospadias [9], [24], [27].

Derived from the pooled data, TVIF onlay and TIP were clinically equivalent for primary proximal hypospadias in terms of complication rate, which ranged from 14.04% [21] to 47.06% [23] and 13.54% [21] to 60.00% [19] for TVIF onlay and TIP, respectively. However, one study (2009 Sujijantararat [24]) observed that TIP had a higher complication rate (37.50%) than TVIF onlay (30.00%) for proximal hypospadias, although without statistical difference. In addition, this figure was obviously higher than the overall complication rate in all meatal positions of TIP (23.53%). Interestingly, another study (2007 Braga [19]) also reported a significant higher rate of fistula after TIP and strikingly difference of fistula locations between TVIF onlay and TIP, with more proximal fistula developing in TIP. This phenomena might be partially explained by the reason that compared to TVIF onlay neourethra, although TIP neourethra has no stricture, its length-to-caliber ratio may be acting as a resistance just beyond the native meatus, which gives rises to a proximal fistula in the vicinity of the original proximal hypospadiac meatus. This explanation was corroborated by the uroflowmetry test and supported by the study of Holmdahl et al [28].

### Implications for Clinical Practice and Research

Although TVIF onlay and TIP urethroplasties were suggested to be comparable for primary proximal hypospadias by our meta-analysis, various factors still needed to be considered before decision-making. For instance, the depth and width of the urethral plate should be assessed. According to a prospective randomized surgical trial, Sarhan et al [29] drew the conclusion that a urethral plate width of 8 mm or greater was essential for successful TIP urethroplasty. Holland et al [30] also reported that urethral fistula after TIP urethroplasty was associated with an initially narrow urethral plate lesser than 8 mm before a relaxing incision. As a result, an alternative repair technique instead of TIP was advocated in proximal hypospadias repair, such as TVIF onlay. Still, a comprehensive assessment for surgical procedure option among cosmetics, functional outcome, complication rate, patient’s condition and surgeon’s preference must be considered in all hypospadias surgery. Actually, both procedures have their fundamental requirements, like a healthy urethral plate and surrounding skin in TIP and a sufficient amount of foreskin for neourethra reconstruction in TVIF onlay.

We advocate that comparative studies of urethroplasties for hypospadias should be present in a universal standard form and more detailed information, especially for preoperative treatment, condition of patients and surgical procedures. For mid shaft and proximal hypospadias locations was proved to come up with significantly different outcomes, the severity of hypospadias and accompanying chordee should be analyzed separately with outcomes in different studies [31]. For instance, if more individual data were available, the comparison of recurrence curvature rate between TVIF onlay and TIP could be analyzed separately by the patients with or without dorsal plication, which would make the results more reliable.

Moreover, objective and quantitative cosmetic and functional evaluation methods need to be employed in addition to general assessments, such as Hypospadias Objective Penile Evaluation [7], [32], PPPS [33] and uroflowmetry, which would facilitate further meta-analysis in this field.

### Limitations

In this meta-analysis, we demonstrated that TVIF onlay and TIP were clinically equivalent for primary proximal hypospadias in terms of postoperative complication. However, this result should be interpreted with caution because some limitations needed to be taken into careful consideration. At first, all included studies were retrospective cohort studies rather than randomized controlled trials (RCT). Secondly, the population of patients was relatively small. Finally, for insufficient detailed data, our results were based on unadjusted estimates, while a more precise analysis would be conducted if individual data were available, allowing for the adjustment by covariates including preoperative treatment and condition, details of surgical procedures, ethnicity, family history and environmental factors.

## Conclusions

This meta-analysis has provided the most comprehensive and reliable comparison between TVIF onlay and TIP urethroplasties up to now, which proves that they are clinically equivalent for primary proximal hypospadias.

Considering limitations described above, more studies about the comparison between TVIF onlay and TIP are needed, especially in large population RCT using standardized and unbiased methods. Also, patients with homogeneity, well-matched controls with standardized outcome analysis and follow-up with sufficient length are preferred. Analysis based on such studies may eventually lead to a better and more precise comparison between TVIF onlay and TIP urethroplasties.

## Supporting Information

Text S1 **Search Strategy.**
(DOC)

Checklist S1 **PRISMA 2009 Checklist.**
(DOC)
