# Supplementary material for: Comparison of Transverse Island Flap Onlay and Tubularized Incised-Plate Urethroplasties for Primary Proximal Hypospadias: A Systematic Review and Meta-Analysis
Source: PLoS One. 2014 Sep 8;9(9):e106917. doi: 10.1371/journal.pone.0106917 (PMC4157843; doi:10.1371/journal.pone.0106917)
Supplement: Text S1 — Search Strategy. (DOC) [file pone.0106917.s001.doc]

Pubmed:

(hypospadias OR hypospadias [mh]) AND (snodgrass OR TIP OR tubularized incised plate OR tubularized incised-plate) AND (transverse island flap onlay OR TVIF onlay OR onlay island flap OR onlay flap)

EMBase and the Cochrane Libraty:

(hypospadias) AND (snodgrass OR TIP OR tubularized incised plate OR tubularized incised-plate) AND (transverse island flap onlay OR TVIF onlay OR onlay island flap OR onlay flap)

Language: English
